# P29 A retrospective multi-centre review of antimicrobial usage in patients diagnosed with aspiration pneumonia

**DOI:** 10.1093/jacamr/dlac004.028

**Published:** 2022-02-16

**Authors:** Blend Ashtey, Oliver Troise, Stephen Hughes

**Affiliations:** Antimicrobial team, Pharmacy department, Chelsea and Westminster NHS Foundation Trust, London, UK; Antimicrobial team, Pharmacy department, Chelsea and Westminster NHS Foundation Trust, London, UK; Antimicrobial team, Pharmacy department, Chelsea and Westminster NHS Foundation Trust, London, UK

## Abstract

**Background:**

Over-diagnosis of infective aspiration pneumonia is common in healthcare settings and represents a potential for misuse of antimicrobial therapy.

**Methods:**

A retrospective analysis was undertaken of all hospitalized patients treated for aspiration pneumonia within a multicentre Acute NHS Trust (April–May 2021, London, UK). Data collected included: age, initial antibiotics prescribed, duration of therapy, relevant microbiology (sputum samples or blood cultures) and chest X-ray. Treatment response was analysed including body temperature after 24 h of antimicrobials, use of oxygen support after 48 h, any escalation of antimicrobial therapy and 30 day in-hospital mortality. The study was registered as service evaluation project.

**Results:**

In total, 94 patients treated for aspiration pneumonia were included; median age 82 years (IQR 67–89). Co-amoxiclav monotherapy was the most frequently prescribed treatment (67/94); cephalosporins (8/94) and ciprofloxacin (4/94) were also commonly used. Duration (median) of treatment was 4.4 days (IQR 1–6.7); 32/94 patients received <48 h. Radiological evidence of consolidation, O_2_ support at 48 h and fever at 24 h was present in 32/94, 25/94 and 12/94 patients, respectively. A microbiological diagnosis to confirm infection was present in 12/94. Consolidation [OR 2.36 (95% CI 1.04–10.25); *P = *0.037] and O_2_ support at 48 h [OR 3.22 (95% CI 1.02–10.25); *P = *0.044], but not fever at 24 h [OR 0.94 (95% CI 0.18–4.80)], were associated with treatment escalation. No association between O_2_ support/fever and total duration was evident. The 30 day in-hospital mortality was 17% (16/94).

**Conclusions:**

Aspiration pneumonia has high in-hospital mortality and often necessitates antibacterial treatment. The true incidence of bacterial pneumonia is unclear, and many patients may benefit from early cessation of antibacterial treatment.

